# Cases of Strangulated Femoral Hernia, in the St. Mary-Le-Bone Infirmary; under the Care of Mr. Stafford

**Published:** 1833-03

**Authors:** 


### HERNIA.

### *Cases of Strangulated Femoral Hernia, in the St. Mary-le-bone Infirmary; under the care of* Mr. Stafford.

Case I. Eliza Robinson, æt. seventy, (a washerwoman,) of the middle stature, and ordinarily having good health, was admitted into St. Mary-le-bone Infirmary on the 4th of October, 1832, for a femoral hernia, which had been strangulated, as she stated, forty-eight hours. She had been subject to a rupture for twelve years, but never till now had there been any difficulty, when the bowel came down, of returning it. The tumor is about the size of a small orange, tense, irregular on its surface, tender on pressure, and is situated on the right side, under Poupart's ligament, in that space on the thigh which is immediately below the crural or femoral ring. The symptoms which she laboured under on her admission were, violent sickness; extreme pain and tenderness of the abdomen; an anxious countenance; a furred tongue; pulse 110, hard, small, and wiry; and, latterly, a constant vomiting of stertoraceous matter.—Ft. V.S. ad ℥xvj. (Blood cupped and buffed) warm bath, in which the taxis was carefully employed. Afterwards a tobacco injection (ʒss. to Oss.), and ice applied on the tumor. No beneficial effect was produced. The operation was performed at two P.M.

An incision was made one inch above Poupart's ligament, extending over the centre of the tumor to about two inches and a half outwards to the lower part of it. The fascia was divided, and the hernial sac exposed. What appeared to be the proper sac was opened, and a quantity of fluid (about half an ounce to one ounce) escaped. There were several adhesions, and it was with difficulty a director could be inserted under the membrane, which was divided up to Poupart's ligament. There was immediately beneath this apparent sac, a membrane covered with fat, and resembling omentum. The femoral ring could not be discovered, which, together with the adhesions, and the general appearance of things, led to the conclusion (for the part exposed had the appearance of intestine) that the sac itself was not opened. For this reason, therefore, what appeared to be intestine was taken up between the finger and thumb, and it was discovered that the gut still lay beneath it. Another opening was cautiously made into this apparent sac, and immediately a tablespoonful of fluid made its escape. As in the last instance, there were firm adhesions, and the same difficulty occurred in passing the director under the divided membranes. There was still reason, as the femoral ring could not be felt incarcerating the gut, to suspect that the proper sac was not yet entered. The part beneath was again pinched up between the finger and thumb, and cautiously opened. A slight quantity of fluid escaped, and the intestine lay immediately below. The *proper* sac was now laid open completely up to the femoral ring, and the stricture causing the strangulation easily felt. A director was inserted, Gimbernat's ligament divided, and the gut, which was of a dark red colour, immediately returned.

The wound was dressed after the usual manner. Half an hour after the operation, she had seven or eight copious motions. Countenance less anxious; pulse ninety; tongue dry and furred. Still felt considerable pain in the abdomen.

Half past three P.M. Ordered twelve leeches to be applied on the abdomen; a fomentation.—Haust. Efferves. quartâ quâque horâ sumend. Fever diet.

Ten P.M. Pain of the abdomen much relieved; but there was tympanitis; pulse ninety, and rather feeble. To continue the effervescing draughts, and to apply a bran poultice over the whole region of the abdomen. In addition to fever diet, a pint of beef-tea.

5th, eight o'clock. Had some sleep during the night: pain subsided, but tympanitis the same. Pulse ninety, soft; tongue dry. To continue the medicine and bran-poultice.

One P.M. Was seized with faintness and difficulty of breathing. Pulse seventy, and hardly perceptible.—Ordered ℞. Sp. Æther. Sulph. ♏xxv.; Sp. Lavand. comp. ♏xxv.; Syrup, ʒss.; Aq. Menth. Virid. ℥x. M. secundis horis s.

Four P.M. Faintness much relieved. No pain; pulse eighty, and stronger.

6th. Had a tolerable night. Bowels had acted once. Pulse eighty, and firm; tongue not quite so dry; tympanitis the same. Ordered an emollient clyster.

Two P.M. Clyster produced one copious motion. Tympanitis rather increased, and the form of the ribs, distended with air, can be distinctly seen. Ordered, ℞. Magn. Sulph. ℥i.; Sp. Æther. Sulph. ʒss.; Mist. Ammon. Acet. ℥xi. M. fiat mist. sumend. ℥ss. 4tis horis.

7th. Bowels had been relieved twice. Good night; and in other respects much the same as yesterday. To go on in the same manner. The dressings were removed, and the wound was found to be united along its whole course, excepting for about half an inch at its upper part.

8th. Much the same as yesterday; tympanitis somewhat less; bowels had not been relieved; the wound looking well. Ordered ℞. Pil. Hydrarg. gr. iv.; Pulv. Rhæi. gr. vj. fiat pil. ij. h. s. s. ℞. Ol. Ricini, ℥ss. cras primo manes.

9th. Better in every respect than yesterday. Pulse seventy, but feeble; tongue moist, and less furred; wound healthy, but its lips had reopened between the ligatures. Ordered, ℞. Pulv. Cinchonæ, gr. xi.; Pulv. Zingiberis, gr. v.; Decoct. Cinchonæ, ℥iss.; Sp. Tnuioris, gtt. xxv. M. quartis horis s. Diet, fish; one pint of porter; red wine, two ounces.

10th. Considerably improved: tympanitis nearly gone; pulse and tongue almost natural; slight inflammation around the margin of the wound. The ligatures were cut and taken away, and the wound brought together by adhesive plaster. To continue the medicine; wine, one gill; and, in addition to the diet, one mutton chop.

From this time the patient progressively went on well: the wound healed on the 6th of November, and, when she could bear the pressure of a truss, she was discharged from the hospital.

*Remark.* The sac being divided into three layers, and containing fluid between each of them, is an extremely curious fact, and may be considered as almost an anomaly. It points out to us that there is no peculiarity which may not present itself in the operation for hernia, and that, whatever general rules may be laid down, we ought to be prepared for every deviation that may occur. This case is also interesting, by its showing the necessity of supporting the patient, both by diet and the administration of tonics, as soon as possible.

Case II. St. Mary-le-bone Infirmary. On November 11th, 1832, Frances Row, æt. sixty-six, a hard-working woman, and of a spare habit, was admitted into this hospital, with a strangulated femoral hernia. The tumor was about the size of an egg, very tense, and tender to the touch, and was accompanied with constant vomiting, and extreme pain and tenderness, on pressure, of the whole region of the abdomen. The intestine had been incarcerated twenty-seven hours. Bleeding, the warm bath, the taxis, and all the usual means, were employed without effect. The operation was performed at twelve P.M., and nothing remarkable was observed, excepting that, when the sac was opened, there was not more than a teaspoonful of fluid in it. When, however, the femoral ring was divided at Gimbernat's ligament, about a quart of serum immediately rushed down from the cavity of the peritoneum, and continued to flow so freely that the wound could not be dressed with adhesive plaster. Two ligatures were consequently employed, and a graduated compress, made with lint, bound down with a bandage upon it. After the operation, she was carried to bed, where she remained quiet for three hours, without taking any medicine. As the bowels had not then acted, and there was considerable pain in the abdomen, she was ordered, ℞. Magn. Sulph. ʒi.; Aq. Menth. Pip. ℥i. M. fiat haustus, omni horâ donec alvus responderit sumendus. App. hirudines xx. region. abdom. App. fotus comm. region. abdom. Diet, fever.

One P.M. Bowels had acted four times. Pulse ninety, and soft; tongue dry and furred.

Three P.M. Seized with very severe pain in the region of the heart, (an attack she had been before subject to,) accompanied with dyspnœa and faintness; pulse 130, feeble, and very irregular. Ordered, ℞. Liq. Opii Sedat. ♏xv.; Sp. Æther. Sulph. ♏xxv.; Mist. Camphora, ʒx. M. statim s.

Four P.M. Somewhat relieved.—℞. Mist. Æther. comp. ℥i. 2dis horis s.

Ten P.M. Much relieved: pain almost gone; pulse 100, regular, but feeble.—To continue the medicine during the night.

13th. No pain. Bowels had not acted.—Emuls. Ol. Ricini, ℥ij. statim s. Operated four times.

14th. Better: no medicine required.

15th. Had had no sleep during the night, but was no worse. Pulse 100, and soft.

Vespere. ℞. Liq. Opii Sed. ♏xxx.; Mist. Camph. ℥i. M. h. s. s. Rep. Haust. Ol. Ric. cras primo mane s.

16th. Had slept well; pulse ninety, and low. After the bowels had acted, to take, ℞. Cinchonæ pulv. gr. xij.; Zingiberis pulv. gr. v.; Decoct. Cinchonæ, ℥iss.; Sp. Ammon. A. gtt. xxv. M. quartis horis s. Diet, one mutton-chop; wine, one gill; porter, half-pint.

The case, from this time, with the exception of a slight return of the pain in the abdomen, (for which leeches were once applied,) went on favorably. She was enabled to take nourishing food, and gathered strength under the tonic treatment. The wound soon healed; but she could not wear a truss as soon as she otherwise might have done, in consequence of the cicatrix remaining tender. She was discharged from the infirmary quite well.

*Remark.* The remarkable points of this case were the sac containing so little fluid, while so large a quantity (showing the completeness of the strangulation of the gut,) rushed through the wound from the cavity of the peritoneum. It shows also, as in the last case, the benefit derived from the early administration of tonics and nourishing food.

CASE III. Elizabeth Appleby, æt. fifty-three, was admitted into St. Mary-le-bone Infirmary, at one P.M. on 3d December, 1832, with a large tumor, situated on the right thigh, immediately below Poupart's ligament, about the largeness and shape of a moderate sized melon. The history she gives of it is as follows: about eight years ago, she was operated upon for a strangulated femoral hernia, and then was advised to wear a truss, and cautioned never to let the rupture come down. At first, with the exception of once or twice, she was enabled to do this; but latterly, within the last two years, she has been obliged to work very hard, in consequence of which, the truss became ineffectual, and she could not prevent an occasional protrusion of the bowel. At these times the tumor presented has been very great, but not so large as at present, and, by lying down on the back, it has generally, of its own accord, gone up again in a few hours. She states that the day before her admission, she took a very long walk, which fatigued her extremely, and during which the rupture came down, and has remained so ever since. She is now suffering great pain in it, as well as in the abdomen; is constantly vomiting, and having also a quick hard pulse, dry tongue, and a distressed anxious countenance.

At two P.M. She was placed in a warm bath; and, when faintness was produced, the taxis was employed with the greatest care. No impression, however, could be made upon the tumor: it remained of the same size, and, as so much pressure might increase the inflammation, by which the chance of success of the operation, if necessary to be performed, would be rendered less, she was carried to bed, and other remedies employed. Ordered Enema Sodæ Sulph. immediately, and the application of ice on the tumor.

Nine P.M. Just the same in every respect. A tobacco (ʒss. to lb ss.) injection. The taxis again carefully employed.

Eleven P.M. All the remedies had failed, and it was found necessary to propose the operation, as the only chance of saving her life. She would not, however, after every persuasion had been used, consent, saying that, when purgatives had been administered on former occasions, it had always gone up, and she had no doubt, if they were employed, it would do so now. She was consequently ordered, Ol. Ricini, ʒiv. quartâ quâque horâ s. To continue the application of ice for an hour or two.

4th, eight o'clock. The bowels had not acted; extremely miserable night; violent pain in the tumor and abdomen; sickness the same; pulse 130, tongue dry and cracking, with constant thirst.

Two P.M. The pain in the tumor has considerably increased. It is larger even than yesterday, in consequence, most probably, of the contents of the incarcerated intestine having been decomposed; and thus, air having been disengaged, it has become more distended. It is now extremely hard and shining, and of a purplish cast, from venous congestion. It occupies the whole space from the pubes to about two inches from the spine of the ilium, and, on its inner side, it extends itself into the right labium, and its upper extremity tilts itself over, and is considerably above Poupart's ligament: in fact, as it has been described before, it resembles a large melon. The patient, in the last extremity, consented to the operation; which was accordingly performed at four o'clock P.M.

An incision was made about three inches in length, longitudinally, on the upper part of the tumor, leaving the lower part untouched. The sac, which lay at least half an inch beneath, in consequence of the œdematous and thickened state of the integument covering it, was opened, and a considerable quantity of fluid escaped. A large mass of intestine, inflated with air, apparently about two yards in length, was exposed. The sac was entirely divided up to the femoral ring, and the strictured part, which prevented the reduction of the hernia, appearing to be chiefly at Poupart's ligament, was cautiously divided by Cooper's knife. An attempt was made to return the intestine, but without success: Gimbernat's ligament was, therefore, divided, and the strictured ring was now freely open. At this time, (as, indeed, had been the case during the whole operation,) the patient was violently retching. The diaphragm and the abdominal muscles, therefore, were in constant action. From this cause there was great difficulty in returning the bowel into the cavity of the abdomen; for, as fast as it was thrust in, the action of these muscles forced it out again. The patient at length began to be faint, and they became passive. The gut, the peritoneal coat of which was highly reddened, was now easily returned. The wound was dressed in the usual manner, and a firm thick graduated compress was laid over it. The patient was carried to bed, and left quiet for three or four hours.

Eight P.M. She was suffering great pain in the abdomen, with extreme tenderness; the sickness had abated; pulse ninety-six, hard and full. Ordered, Fiat V. S. ad ℥xxiv.; Fot. abdomin. ℞. Magn. Sulph. ʒi.; Inf. Rosæ, Menth. Pip. *aa* ℥ss. M. omni horâ sumendus donec alv. respdt.

Ten P.M. Pain still considerable: bowels had operated three times. Ordered, App. Hirudines xx. region. abd.; Cataplasm. furfuris.

5th. Pain nearly gone; pulse one hundred, and soft; tympanitis; tongue dry; extreme thirst. Ordered, Haust. Efferves. quarta quâque horâ s.

6th. Had a tolerable night. No pain; tympanitis the same; pulse ninety, and not so strong; tongue the same, and thirst still great. To continue the effervescing draughts. In addition to the diet one pint of beef-tea, and two ounces of red wine.

7th. Much the same as yesterday. Ordered, ℞. Mist. Cinchon. arom., Aq. Menth. Pip. *aa* ℥ss. M. quartâ quâque horâ sumendus.

8th. Still weaker; in other respects much the same. Occasionally feels faint, and is very restless and oppressed. ℞. Mist. Cardiac. ℥iss. quarta quâque horâ s. ℞. Liq. Opii Sedat. ♏ xxv.; Mist. Camph. ℥i. M. fiat haust. h. s. s.

9th. Much worse; diarrhœa, with extreme lassitude, pulse ninety, and feeble.

From this time she gradually sunk, and died on the 12th, being ten days from the time the operation was performed.

On examination of the body twenty-four hours after death, the thoracic viscera were found healthy. The liver, the stomach, and spleen, were healthy. Two yards and a half, by measurement, of the small intestines were blackened, and in a semi-mortified state, not entirely having lost their toughness, and yet could be torn by a little force. The mucous surface was ulcerated, and the peritoneal coat, in various parts of it, was thickly covered by purulent lymph. A large semi-cartilaginous tumor, about the size of a citron, was found in the uterus; and the other parts were healthy.

*Remarks.* This case points out to us the necessity of performing the operation as soon as possible; for, had this patient submitted to it when it was first proposed to her, it is most probable she would have recovered. The immense portion of intestine contained in the sac is, perhaps, unparalleled in femoral hernia, and it can only be accounted for by the operation having been performed before, in consequence of which the femoral ring was enlarged beyond its natural size. The difficulty of returning the gut arose partly from its great bulk, partly from its being distended with air, and partly from the constant action of the diaphragm and abdominal muscles: had the intestine been unreturnable, it would have been advisable, perhaps, to have punctured it with a needle, and let out the air, when it would have been easily effected.

Case IV. St. Mary-le-bone Infirmary, December 23d, 1832. Ann Baker, æt. forty-nine, an emaciated, unhealthy-looking woman, states that she has had a rupture for several years, and has constantly worn a truss for it. In spite of this instrument, however, a portion of intestine has occasionally protruded itself through the ring, but has always gone back again without difficulty. According to her own account, the bowel has now only been strangulated four hours, but, from the previous symptoms and the state of the part when operated upon, it is probable it has been down for a longer period. On Saturday she felt pain in the part; and on Sunday, the day of her admission, (about one o'clock,) she was seized with constant sickness, which she attributed to having eaten pork, and which continued without abatement until the present time, (half-past ten P.M.) The tumor is about the size of a turkey's egg, elongated from the pubes towards the anterior superior spine of the ilium, is extremely tense, very painful and tender on being touched, and tilting over Poupart's ligament. Her present symptoms are, extreme pain in the abdomen, with tenderness on pressure; constant retchings; anxious countenance; pulse ninety-six, hard, and wiry; and a dry furred tongue.

She was bled to sixteen ounces; placed in the warm bath; the taxis employed; and purgative injections thrown up the rectum; but the tumor remained of the same size. The injection brought away a large quantity of feculent matter; in consequence of which, (about half-past one in the morning,) she was again placed in the warm bath, and the taxis again employed No favorable result ensued; the tumor remained as tense and painful as before, the sickness and pain of the abdomen continued. The operation was consequently performed.

An incision was made, by pinching up the integuments over the tumor, and thrusting a double-edged scalpel through it at its base and cutting out, immediately opposite to the centre of the femoral ring, and extending to about two inches in length. The parts were cautiously dissected down to the sac, which was opened and divided up to the femoral ring. The sac contained a large piece of omentum, and a dark chocolate-colour knuckle of intestine, which was of about three inches in length, lying immediately beneath the latter. The stricture was divided at Gimbernat's ligament, and the gut easily returned. The omentum, however, was firmly adherent to the mouth of the sac. An attempt was made to break down the adhesions, some of which yielded, but the whole of them could not be torn through. As the patient was not in a state to lose much blood, in consequence of having a disease of the heart, and as the portion of the obtruded omentum was supplied by several large vessels, which it might be difficult to tie, a ligature was tightly placed round it at its base, and it was then cut off. The separated portion of omentum measured in breadth four inches and three fourths, and in length three inches. The wound was brought together by one ligature and adhesive plaster, and a compress was applied.

Immediately after the operation, the sickness was relieved, and the pain gradually subsided: she was seized, however, with a slight rigor. ℞. Liq. Opii Sed. ♏xxx.; Mist. Camph. ℥i. M. fiat haust. statim s.

24th. She had had, during the night, three pretty copious motions; is free from pain; no sickness, and disposed to sleep.

Six P.M. Felt severe pain on the right side of the epigastric region with tenderness; pulse ninety, hard and wiry. Ordered App. Hirudines xv.

Eleven P.M. Was much exhausted from the leeches. Pulse 120, very feeble and indistinct; pain relieved. Ordered Enema emolliens statim inj. ℞. Ammon. Carb. gr. v.; Sp. Æther. Sulph. m. xij.; Mist. Camph. ℥i.; Tinct. Amm. C. ʒi. M. fiat haust., quartâ quâque horâ sumend. Wine two ounces, to be taken occasionally in water.

25th. Had slept well during the night. Now and then felt slight pain in the abdomen; pulse one hundred, stronger, and more distinct; tongue moist and slightly furred. Ordered Haust. Ol. Ricini. statim s. Foment. commun. abd. Rep. Enema.

Three P.M. Feels pain in the abdomen; tender on pressure. App. Hirudines xij. abdomen. Rep. Haust.

Ten P.M. Much relieved. Feels very faint and low; pulse 115, very feeble, and indistinct. Bowels had acted four times. Ordered one ounce red wine in arrowroot, some of which to be taken occasionally during the night. Rep. Haust. Ammon. Carbon. ℞. Ext. Papav. alb. gr. vj.; Pil. Hydr. gr. iv. fiat pil. ij. statim s.

26th. Had a very comfortable night, having slept several hours; no pain; pulse one hundred, more full and distinct; tongue moist and less furred. Wound dressed and looking well. Ordered, ℞. Pulv. Cinchonæ, gr. xi.; Pulv. Zingib. gr. vi.; Dec. Cinch. ℥iss.; Sp. Tenuioris, gtt. xxv. M. quartâ quâque horâ sumendus. Rept. pil. h. s. s. Beef-tea, one pint; wine, two ounces, daily.

From this time the case went on favorably.

27th. Much better; had a good night; no pain; bowels relieved three times; enjoyed the beef-tea, and feels stronger; pulse one hundred, and firmer. To continue Haust. Cinchon. Arom. et Pil. Papav. h. s. s. Wound looking well, but the tied omentum not come away.

28th. Going on well in every respect, excepting having retention of urine. Catheter to be passed night and day. To go on in the same manner. Three pints of beef-tea.

30th. Going on favorably; pulse ninety; tongue moist and clear. To continue medicine. In addition to the present diet, two eggs daily, four ounces of fish.

31st. Still improving; pulse eighty-four. Ordered one mutton-chop, half a pint of porter. Cut surface of the omentum granulating, and filling up the femoral ring. On the following day, the diet was increased to two mutton-chops, one pint of porter, one gill and a half of red wine; she continued the bark. The ligature was taken away on the 13th, and the wound was healed on February 2d, the granulations of the cut surface of the omentum, and those of the wound itself, having united and skinned over.

*Remarks.* It has been a disputed point among surgeons, when the omentum cannot be returned, whether it should be cut off or the vessels tied, or whether a ligature should be placed at its base, and then cut away? The general opinion is, that it should be removed, and the vessels tied, with the view of preventing subsequent inflammation of the peritoneum, and the possibility of secondary hemorrhage. In the case before us, it was not advisable, because, if any difficulty had arisen in tying the vessels, so much blood might have been lost, that the patient, having a disease of the heart at the time, and a very feeble pulse, might have sunk under the operation. It shows, therefore, although the application of a ligature on the omentum may be against the generally-received opinion, that it may be done under peculiar circumstances, without much apprehension of future evil consequences.

---

